# Evolutionary medicine of emunctory functions of the kidney: an empirical review

**DOI:** 10.1093/emph/eoaf019

**Published:** 2025-08-05

**Authors:** Noel T Boaz, Robert L Chevalier

**Affiliations:** Center for Evolutionary Medicine, Integrative Centers for Science and Medicine, Martinsville, VA, USA; Virginia Museum of Natural History, Martinsville, VA, USA; Department of Biology, Norfolk State University, Norfolk, VA, USA; Department of Pediatrics, University of Virginia, Charlottesville, VA, USA

**Keywords:** kidney, renal-evolution, kidney-disease, evolutionary-medicine

## Abstract

Primitive emunctory functions to expel harmful substances from cells and the interstitial space of multicellular organisms evolved over the past billion and a half years into the complex physiology of the metanephric kidney. Integrative biology allows empirical testing of hypotheses of the origins of renal structures from homologous single-celled precursors. Emunctory cell complexes called nephridia evolved in metazoan (cnidarian) ancestors 750 million years ago (mya). The pronephric kidney was a metameric structure that evolved some 700 mya in early bilaterians to excrete waste products through nephridial slits in the body wall from head to tail. The mesonephric kidney evolved 635 mya when pharyngeal slits differentiated into filter-feeding gills and a heart-kidney evolved in later bilaterians. The mesonephric filtering glomeruli lost their external exits through the body wall and now drained through an internal mesonephric duct into the coelom. When chordates moved into fresh water from the sea 588 mya the high-pressure glomerulus evolved in the mesonephros, increasing water excretion. Tetrapods moved onto land losing the buoyancy of water. Blood pressure and glomerular filtration rose and the metanephric kidney evolved in amniotes. The high pressure-flow glomerulus predisposes podocytes to injury and detachment leading to sclerosis, whereas the high mitochondrial activity of the tubule contributes to susceptibility to ischemia, hypoxia, and oxidative injury. The kidney evolved a counter-current mechanism and urea cycle to optimize water retention. Perturbations in the complex development of the metanephric kidney, which parallels its phylogeny, explain many renal pathologies, which are traceable to these adaptations.

## INTRODUCTION

Chronic kidney disease has become one of the leading causes of global morbidity and mortality, but effective therapies are limited by the vulnerability of the kidney to both hemodynamic and metabolic injury [1]. The ultimate causes of vulnerability of this excretory organ lie in its long evolutionary history and its adaptation to a changing planetary environment [2, 3]. In the course of a human lifespan the number of nephrons formed at birth is dependent on fetal environment and reduced by 50% by late adulthood, the product of susceptibility of the metanephric kidney to progressive glomerular and tubular injury [4, 5].

Human kidneys basically function to make urine, but as evolutionary physiologist Homer Smith famously intoned, ‘in a more considered view one can say that the kidneys make the stuff of philosophy itself’ [6]. Admittedly grandiose, Smith’s point is that the metabolically expensive kidneys have played an important role in maintaining physiological homeostasis through evolutionary history. In this review we concentrate on the most fundamental of the kidney’s functions, excreting waste products.

Delineating the evolutionary steps that account for kidney anatomy and physiology, and how they were adaptive in their own time and place, is essential to a full understanding of renal function. Darwin does not promise that the gradations must be teleologically linear or that their logic must cohere to our modern-day synchronic notions of efficient or practicable perfection [7]. The human kidney must recover 99% of the filtrate, an energetically expensive and inefficient process. Understanding the multifarious interactions of the kidney in health and disease has been a major biomedical challenge. If we seek to understand the kidney biomedically we must understand its evolutionary history.

In this review we consider the morphophysiological adaptations of the urinary system through time. Homologous genes and comparative biology of extant organisms now inform us of ancient precursors of the kidney that extend back to Proterozoic single-celled precursors. Because this ancestry traces back before the eumetazoan evolution of organs *per se*, virtually every other organ system in the body has been affected by the ontophylogenetic form and function of the kidney, and instructive comparisons may involve unfamiliar organisms. New discoveries in ‘omics fields have potentiated reliable tests of homology, allowing more precise and well- controlled “time tree” phylogenies to be reconstructed [8, 9]. New paleontological discoveries have filled in many gaps in the fossil record, replete with reliable paleoenvironmental data and geochronological control [10]. It is now possible using the methods of Evolutionary and Developmental (Evo-Devo) Biology [11, 12, 13, 14] to elucidate the origins of renal physiology to apply them to clinical concerns within the developing discipline of Evolutionary Medicine [3, 15]. Because the nomenclature of kidney evolution has been variously misinterpreted, we include a glossary of terms as applied in this review.

Evolutionary medicine researches the ultimate or evolutionary causes of pathologies [16, 17, 18, 19, 20, 21, 22, 23]. In this approach human structure and function are understood as products of adaptations honed by natural selection through geological time, at many evolutionary levels and through many different ecologies. This approach is not new (Explanatory Box 1), but we are now able to chart through time the net result of gene cascades and developmental bauplans nested within one another by natural selection over eons. Renal malformation or disease result when evolved renal adaptations go awry: an evolutionary medical perspective can benefit future research in medical and surgical care of these disorders [24].

Box 1. Two Influential Narratives of Kidney Evolution.

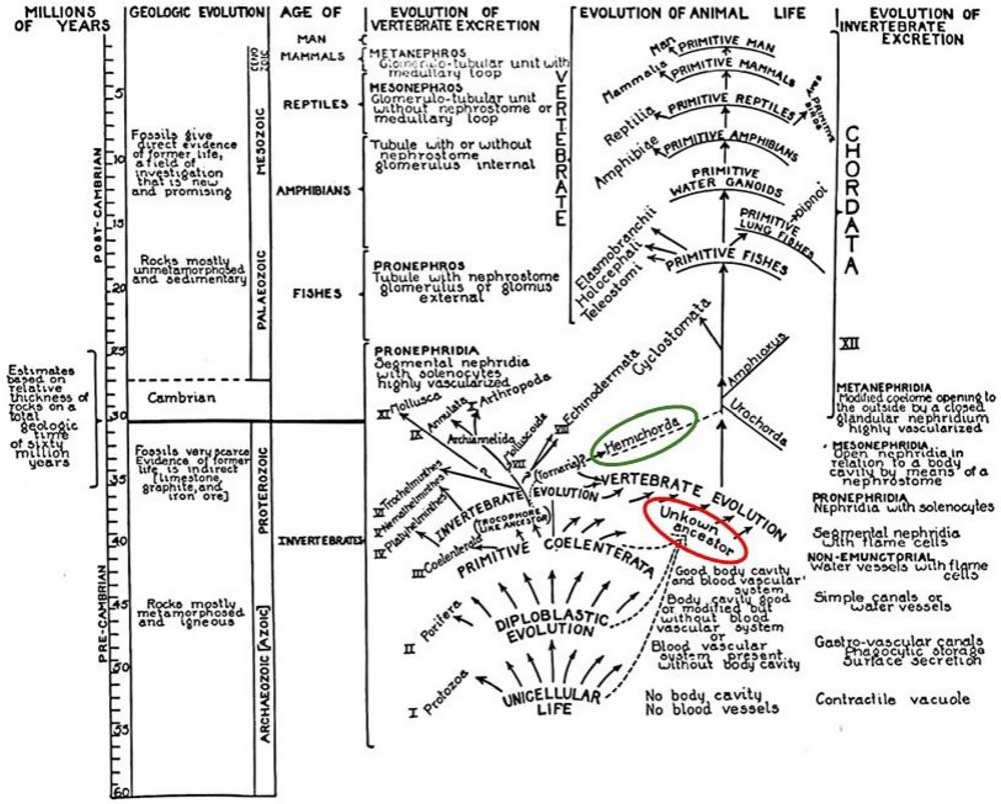

The evolution of excretion outlined by urologist Frank Hinman of the University of California, San Francisco in his 1111-page opus on the *Principles and Practice of Urology* (1937) [25] is shown above. Hinman marshalled comparative anatomical and developmental evidence to posit stages of invertebrate and vertebrate excretory evolution beginning in the Pre-Cambrian, but he refrained from connecting the two. Stymied by a dearth of fossils of soft-bodied ancestors he listed an ‘Unknown Ancestor’ (circled in red) leading from invertebrates to vertebrates. Presciently, however, he figured a hypothetical dashed line (circled in green) from hemichordates to the base of the vertebrate lineage, a relationship that has now been shown to be homologous in the Genomic Age, as discussed in this review.

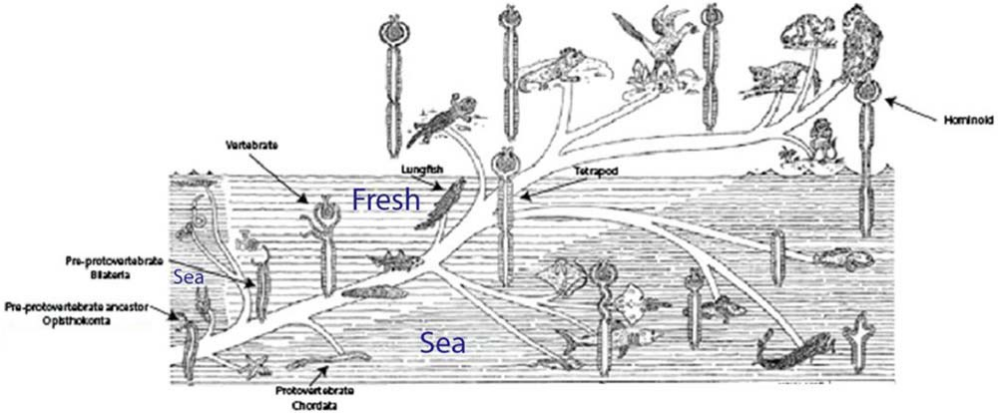

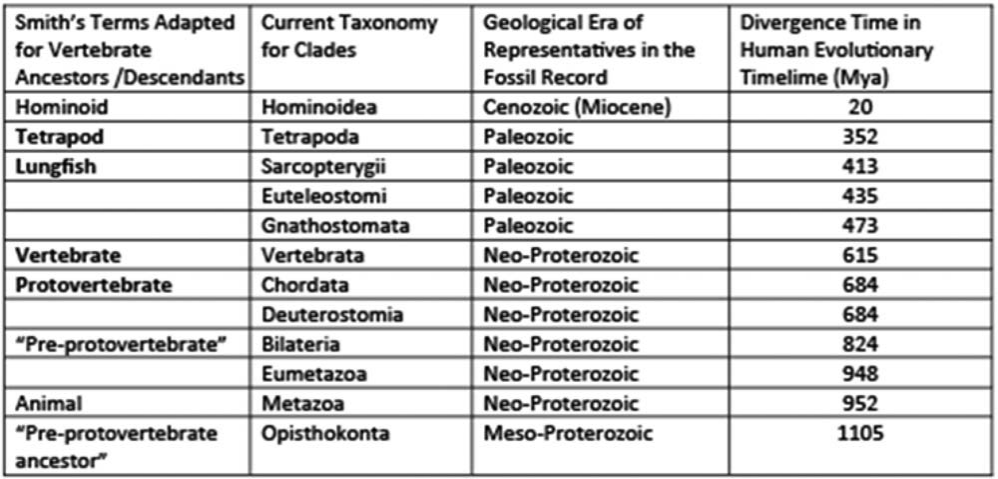

Above is depicted another seminal theory of renal evolution, that of renal physiologist Homer Smith of the University of Virginia and subsequently New York University [26] Smith’s theory focused on the origins of the vertebrate kidney, for which he hypothesized a ‘protovertebrate’ chordate ancestor similar to the extant lancelet, despite a lack of fossil evidence. That hypothesis has since been widely supported by genomic, comparative anatomical, and paleontological evidence. Smith’s formulation was also innovative in including ecological context in his consideration of renal adaptations, most notably in respect to the freshwater origins of the vertebrate kidney. His ideas have been very influential in renal physiology and have continued to be debated to the present day, as discussed in this review. The table above updates terms used by Smith to current zoological taxonomy, geological era, and best-fit evolutionary divergence time as indicated in Timetree.org (see text.).

## THE PHYLOGENY OF RENAL EMUNCTORY FUNCTIONS

The primary and most primitive function of the kidney as an organ is ‘emunctory’ [25], expelling useless or harmful substances via ultrafiltration and secretion, with much the same effect as vacuolization and expulsion of substances across the cell membrane by the earliest cells. Expelled substances were nitrogenous wastes such as ammonia, urea, and urate, and xenobiotic substances such as alkaloids and toxins. At one stage of evolution (between chordates and the first vertebrates, Table 1), low-salinity water became a potentially fatal substance that had to be excreted [6]. Explanatory Box 1 reviews early but still prescient broad evolutionary narratives of kidney evolution.

**Table 1:** Normal adaptations by evolutionary clades in *Homo sapiens.*

Clade	Divergence date (mya)	Divergence geological stage age	Anatomy	Physiology	Ecology	Extant homologue
LUCA/Cellular Organisms 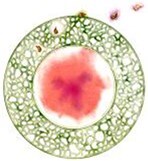	4250	Hadean/ Eoarchean	Single-cell prokayote, bilaminar cell membrane, very small size, ribosomes	Transcription and translation, exocytosis of nitrogenous wastes, Wood-Ljungdahl Pathway, thermophile	Hydothermalvent/hot-spring, anoxic, marine/ ?Freshwater	*Clostridioides*
Eukaryota 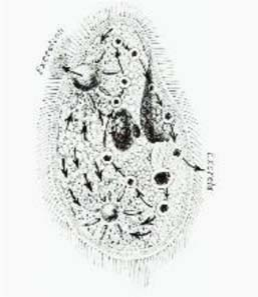	1598	Paleo/Mesoproterozoic	Pronephridia, cell nucleus, mitochondria, vacuoles, ribosomes, cytoskeleton, lysosomes, peroxisomes	TCA Cycle, oxidative metabolism, photosynthesis, ECM genes	Marine	*Paramecium*
Opisthokonta 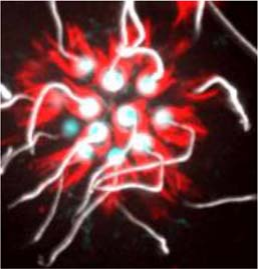	1275	Mesoproterozoic	Hydrostatic skeleton, apical flagellae	Adhesion genes, predatory on bacteria	Marine/freshwater	*Monosiga* (choano-flagellate)
Metazoa 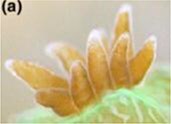	758	Neoproterozoic	Nephridia of 3 cells, radial symmetry, diploblastic, coelenteron, mouth, tentacles, nerve net	Pax genes, ETS, HOX, POU, T-box transcription	Marine	*Acropora* (Basal Cnidarian; larval ‘planula’)
Eumetazoa 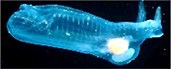	743	Neoproterozoic	Triploblastic, mesodermal muscle, neurons	Glutamate neurotransmitter, ciliated locomotion	Marine	*Thalia* (Salp), Comb jelly
Bilateria Early 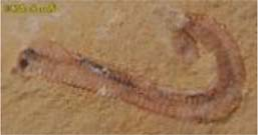 Late 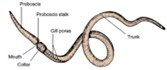	708 635	Neoproterozoic	Pronephros with protonephridia, open circulatory system, protostomal (acoelomate) digestive tract, head and tail in rostro-caudal axis, special sense organs; Coelom, pronephric glomeruli with metanephridia, deuterostomal digestive tract, heart-kidney, closed circulatory system, cloacal excretion, endostyle	Peristalsis, excretory organs, nerve transmission to circular and longitudinal muscle, ammonia excretion. Gills with flitering and respiratory functions, detritus feeder, iodine utilization, transcription factors *eya, six1/2, pou3, sall, ihx1/5* and *osr*, and structural genes *nephrin, kirre, and zo1* AVP precursor/ADH	Marine	*Caeno-rhabditis elegans*/*Priapulus* (Penis worm)/Planaria (flat worm) *Sacco-glossus* (Acorn worm [Hemochor-date])
Chordata 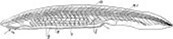	588	Neoproterozoic	Mesonephric glomeruli with metanephridia, pharyngeal filtering gills replace pronephric slits, cartilaginous skeleton, integument, larger body size, thyroid gland	Filter feeder, integumentary respiration, closed circulatory system	Marine/estuarine	*Branchiosto-ma* (lancelet)
Vertebrata 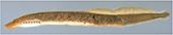	563	Neoproterozoic	Mesonephros with higher-pressure glomerulus, 2-chambered heart, respiratory gills, limbs, post-anal tail, ostracoderm bony skeleton, cranial nerves, parathyroid glands	Gill respiration, Vit. D, AVP V1, V2, increased locomotor speed	Freshwater	*Ichthyo-myzon, Lampetra* (lamprey)
Gnathostomata 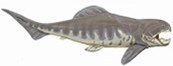	462	Ordovician	Jaws, teeth, separate anal and genital openings, pelvic fins, basipterygia copulatory organs	Urea excretion	Freshwater	*Chimera* (rabbit fish)
Sarcopterygii 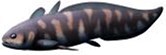	415	Devonian	D7 diluting segment	Urea excretion	Freshwater	*Latimeria* (coelacanth)
Dipnotetrapodo-morpha 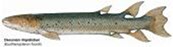	408	Devonian	Lung, suprarenal gland	Mixed gill and air respiration, aldosterone secretion	Freshwater/episodically terrestrial	*Protopterus* (lungfish)
Tetrapoda 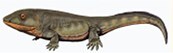	352	Carboniferous	Larval mesonephros, 3-chambered heart, pentadactyly, mesonephric duct connects to testis to become ductus deferens	Lung and integumental respiration, increased basal metabolic rate	Terrestrial with aquatic reproduction	*Xenopus* (clawed frog)
Amniota 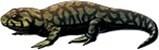	319	Carboniferous	Metanephric kidney, urinary bladder, renal medulla, renal venous portal system	Amniotic egg reproduction, renin-angiotensin-aldosterone axis, increased basal metabolic rate	Terrestrial	*Anolis* (lizard)
Mammalia 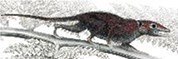	180	Jurassic	Nephron loop (of Henle), high blood pressure, single renal artery, no renal portal system, homeothermy, heterodonty, hair, 4-chambered heart, descent of testes, synepithelio-chorial placenta	Live birth, milk production, increased protection of young, lactation, increased basal metabolic rate	Terrestrial	*Rattus* (rat)
Primates 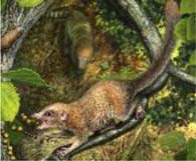	74	Cretaceous	Orbital frontality, prehensile hands and feet, thinned (hemochorial) placenta	Quadrupedal climbing, insectivor-ous/frugivorous requiring Vitamin C	Terrestrial/arboreal	*Chlorocebus* (green monkey)
Hominidae 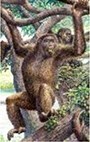	15	Middle Miocene	Increased brain size, increased body size, truncal erectness, loss of tail, facultative bipedality; urate oxidase gene loss	Uricosuria, frugivory	Terrestrial/arboreal (forest/wood-land)	*Pongo* (orangutan)
Homininae 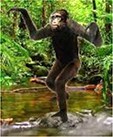	9	Late Miocene	Thermoregulatory adaptations such as sweat glands, loss of hair over most of body (humans), primary bipedality (humans), encephalization (humans)	Uricosuria, brachiation/facul-tative bipedality/knuckle-walking, increased carnivorous diet	Terrestrial (forest/woodland/savanna)	*Pan* (chimpanzee)

Dates of evolutionary divergence are given in millions of years before present. Anatomy, physiology, and ecology of ancestral organisms are listed in columns 4 through 6. Extant/model organisms that show homologous traits and similar adaptations to ancestors are listed in the rightmost column. LUCA art by artist Gaylene Barnes, by permission.

**Figure 1:**
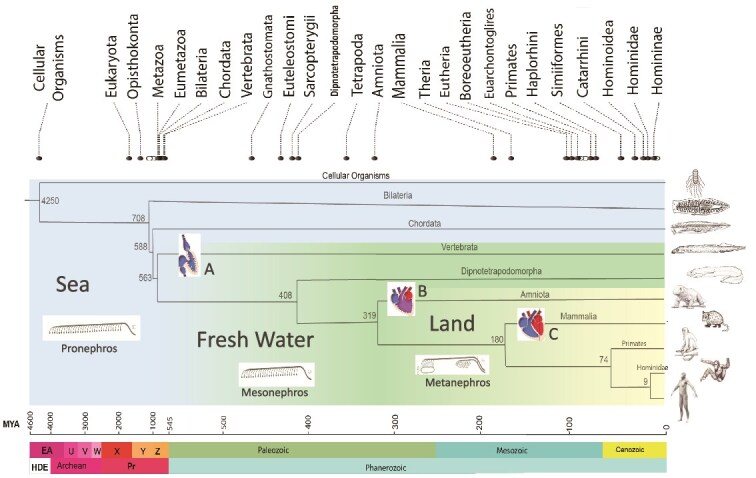
A TimeTree phylogram for *Homo sapiens* back to the last universal common ancestor (LUCA) at 4.25 billion years ago, constructed and reconfigured from Timetree.org. Divergence dates of 30 lineages are indicated by dark circles for named divergences and open circles indicating unnamed divergences. Taxonomic categories are arranged by decreasing rank from left to right at the top of the figure. A phylogeny of 10 selected lineages with best-fit divergence dates in millions of years is shown in the middle of the figure. Separate areas of the phylogeny depict marine habitat (“Sea” shown in blue), fresh water habitat (shown in blue-green), and terrestrial habitat (“Land” shown in yellow). The geological time scale in millions of years ago (MYA) with eras (above) and eons (below) is shown at the bottom of the figure. Abbreviations are: A = 2-chambered heart, B = 3-chambered heart, C = 4-chambered heart, HDE = Hadean, Pr = Proterozoic, EA = Eoarchean, U=Paleoarchean, V = Mesoarchean, W=Neoarchean, X = Paleoproterozoic, Y = Mesoproterozoic, Z = Neoproterozoic.

Figure 1 presents an evolutionary time tree of *Homo sapiens* derived from consensus genomic and paleontological data [27] within a well constrained taxonomic and temporal framework that allows reconstruction of broad paleoenvironmental contexts. Methodology for constructing the phylogenetic diagram used Timetree5 software [9] to create an evolutionary timeline for *H. sapiens* with 30 best-fit adjusted divergence dates derived from molecular and paleontological databases. This perspective grounds our review of renal adaptations. Genomics and integrative biology have allowed a much more precise understanding of the phylogenetic relationships of whole phyla of species [11], especially in the earliest prevertebrate phases of human evolution. These earliest phases of renal evolution, though dimly glimpsed in the last century [6, 25], remain key to understanding the kidney’s adaptations in the surrounding medium of water through time (see Explanatory Box 1).

## EXCRETION OF METABOLIC WASTE PRODUCTS AND FOREIGN CHEMICALS

**Figure 2:**
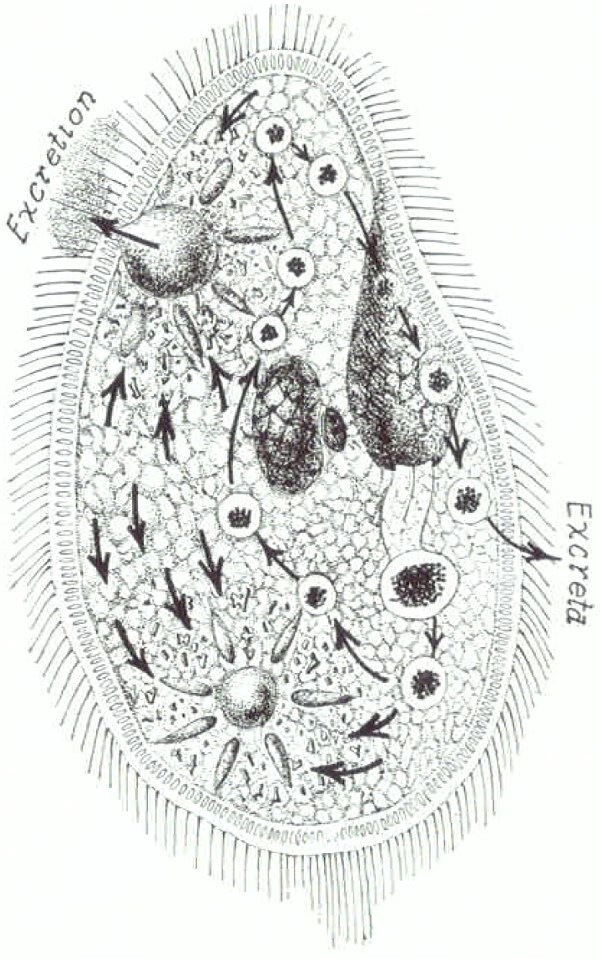
Diagram of a single-celled eukaryotic *Paramecium* showing ingestion of solid food particles and water into its cytoplasm, digestion, and finally extrusion as excreta, shown by arrows to the exterior. Internal circulation of water with uptake of waste products into a cytosolic vacuole, shown by internal arrows, binds with the cell membrane and empties its contents to the exterior. *Paramecium* diverged from the human time tree some 1.6 billion years ago [9]. Figure from Hinman [25].

Our eukaryotic premetazoan, single-celled ancestor expelled metabolic waste products and foreign chemicals by nondiscriminatory ‘emunctory’ processes that included ions, water, acids, and bases. This Last Universal Common Ancestor (LUCA, Table 1) was an early autotrophic (chemolithotropic) organism that lived in the Hadean Eon, estimated at 4.25 billion years ago [10, 27, 28]. Emunctory processes in early cells, emerging some 1.6 billion years ago in human phylogeny, were similar to the extant *Paramecium* (Fig. 2).

Emunctory function entails the trans-membrane diffusion of ammonia or the intracellular engulfing and vacuolation of nitrogenous wastes and xenobiotic substances expelled into the environment by fusion with the cellular membrane. These processes are ubiquitous in single- celled organisms and remain within the realm of functions of individual cells now embedded within multicellular organisms, including the human kidney tubule. Evolution was to elaborate them into the specialized, multi-organ physiological processes of excretion, secretion, digestion, respiration, and circulation [25, 29]. Active excretion across digestive tissues evolved in pre- bilaterian Metazoa (the Cnidaria and Xenacoelomorpha of Andrikou [30]) (Table 1, Fig. 1).

**Figure 3:**
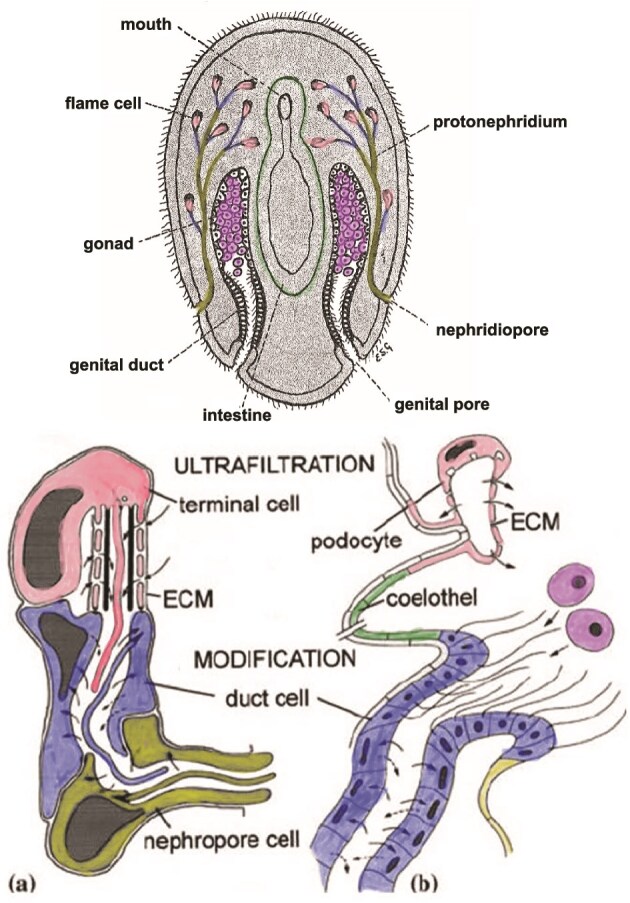
Bilaterian nephridia. Above: Cross-section in dorsal view of a planarian flatworm [27], showing the bilaterian body plan in which the gut is not surrounded by a body cavity (a coelom). The emunctory renal precursor, the protonephridia, drains the large interstitial space directly to the exterior via the nephridiopore. Below: (a) a protonephridium composed of three cells—Terminal (flame) cell, duct cell, and nephropore cell, characteristic of early bilaterians. Solid arrows indicate flow of fluid from the intercellular space through the extracellular membrane (ECM), then reabsorption and secretion by the duct cell, and reaborption by the nephropore cell. (b) the metanephridium evolves in later bilaterians when kidney ultrafiltrate drains into the coelom, and not directly out of the body. The coelom is lined by a mesothelium, termed here the ‘coelothel’. Figure adapted from [31] (below) and [32] (above).

The transition from primitive emunctory to excretory functions in the human evolutionary lineage occurred some 700 million years ago in the basal bilaterian animals, organisms similar to the extant nematode worm, *Caenorhabditis elegans* (Table 1) [31]. Living in sandy near-shore seabeds, these small marine creatures evolved three-cell proto-nephron complexes known as protonephridia (Fig. 3a). Protonephridia used ciliary action to expel internal waste through the body wall to the exterior. A terminal ‘flame’ cell ultra-filtered interstitial fluid through fenestrations in its cell wall and across a specialized extracellular membrane, retaining the larger protein molecules and letting the smaller ammonia and urea molecules flow out with water into a channel formed by the second cell in the series, a duct cell. The duct cell had specialized cell walls that were both resorptive (engulfing and reclaiming small molecules, including water, that the cell still needed), and secretory (removing more small xenobiotic waste molecules from the interstitial space and adding them to the filtrate). The third inline protonephridial unit was the nephropore cell which formed a channel, the walls of which also resorbed water and other small molecules, and then transported excreted waste fluid to the exterior through apertures in the body wall.

Recent genomic studies have shown that regulatory genes expressed in the development of ultrafiltration-based cellular aggregates in a wide array of bilaterians are conserved evolutionarily [33]. Transcription factors *eya*, *six1/2*, *pou3*, *sall*, *ihx1/5*, and *osr*, and structural genes *nephrin*, *kirre*, and *zo1* are homologous throughout Bilateria. This evidence supports the hypothesis that the last common ancestor of bilaterians, dating to ca. 708 Ma (Table 1, Fig. 1), possessed renal precursor cells that could selectively filter out by size some molecules and retain other molecules to maintain internal homeostasis. This protonephridial adaptation was homologous and broadly ancestral to later invertebrate and vertebrate macroscopic excretory organs (Fig. 3b). The terminal cell evolved into a fenestrated cell termed the podocyte which forms the filtration slit of the metanephric glomerulus [34]. The podocytes form the visceral walls of glomerular (Bowman’s) capsules. Parietal walls originate as embryonic vesicles from intermediate mesoderm (35) or phylogenetically as detached segments of the coelomic membrane [31, 35].

### The pronephros

The importance of an Evo-Devo approach to kidney evolution is underscored by the sequential appearance in the human embryo of a transient nonfunctioning pronephros, followed by a transient functioning mesonephros, followed by the metanephros that begins to excrete urine by the 10^th^ fetal week. Induction of the mesonephros is dependent on the formation of a rudimentary pronephros, and induction of the metanephros requires prior formation of the mesonephros.

Evidence now supports that previously scattered protonephridial cells massed together in bilaterians to form an elongate modular organ, the primordial kidney, which we term here, based on genomic and comparative anatomical evidence, the ‘pronephros.’ Romer [36] termed the largely hypothetical primordial kidney of vertebrates the ‘holonephros,’ a term that has fallen into disuse, but he identified it with the pronephros, the embryonic ‘fore-kidney.’ Supporting an even earlier renal primordium, Gasiorowski et al. [33] stated that ‘[o]ur results show that all types of ultrafiltration-based excretory organs are patterned by a conserved set of developmental genes, an observation that supports their homology. We propose that the last common ancestor of protostomes and deuterostomes [i.e. Bilateria] already possessed an ultrafiltration-based organ that later gave rise to the vast diversity of extant excretory organs, including both proto- and metanephridia.’

The pronephros then was an excretory structure extending from head to tail (Fig. 1). It is an ancient structure in human ancestry, appearing only transiently in human embryological development [37], but retaining its plesiomorphic anatomy in some extant primitive bilaterians such as priapulids (‘penis worms’) [31]. These species show protonephridial openings on the exteriors of their bodies. In human embryology only the cervical portion of the pronephros (the ‘fore-kidney’) is observed as a discrete structure, whereas the middle and caudal portions of the pronephros (termed by Romer [35] the ‘opisthonephros’) become incorporated during development into the mesonephros (‘middle kidney’) and metanephros (the ‘great kidney’). Because of this preemption of the middle and caudal segments of the pronephros by subsequent evolution, the embryological literature has tended to treat the pronephros as a cervical rudiment only. Comparative anatomy and genomics have now rectified this perspective, and the pronephros is treated here as a modular renal structure extending the cranio-caudal length of early bilaterians.

In the early bilaterians there was not yet a coelom surrounding the now unidirectional gastrointestinal system from mouth to anus and there was no heart and circulatory system (as seen in the extant planarian flatworms and penis worms), so the common description of the pronephric nephron as ‘a vascular glomerulus protruding into the coelom’ [38] is no longer accurate. The pronephros nephridial slits lost their connections to the pronephric duct internally, connecting instead to the nearby cranial portion of the gut tube, the future pharynx [39]. They retained their primitive nephridiopore outlets to the exterior of the body. These openings became gills, a filter-feeding adaptation that trapped plankton and detritus from gulped water that was then pumped out through the filtering membrane of the pharynx by muscle contraction, with the retained food particles then swallowed.

The pronephros postdated the evolutionary divergence of all bilaterians and predated in human phylogeny the anatomical adaptations now recognized as first arising in hemichordates, exemplified by the extant acorn worms [40] (Fig. 3, Table 1). Filtrate flowed from the interstitium to a nephrostome (ciliated duct cell, Fig. 3b), a ciliated tubule that drains into the proximal nephric tubule, distal tubule, and pronephric duct to the exterior of the body (i.e. not into a nonexistent coelom). There are four tubule domains in the developing pronephros of the African clawed frog: proximal, intermediate, distal, and connecting tubule segments, with close homologies with the human metanephric tubules [41]. The proximal tubule expresses transporters for glucose, amino acids, and peptide secretion of organic acids and anions. The distal tubule shares homologies with the metanephric thick ascending limb of Henle’s Loop (impermeable to water and active reabsorption of sodium) and distal convoluted tubule (regulation of pH and ammonia transport) [40]. In the larval axolotl the pronephric duct epithelium consists of one cell type with basolateral Na/K ATPase for sodium reabsorption and presumably also contains separate apical channels for potassium secretion into the filtrate and sodium uptake from the filtrate [42], indicating homology with the vertebrate collecting duct.

There is no evidence of a juxataglomerular apparatus in the early bilaterian pronephros, which evolved later in association with the developing cardiovascular system.

**Figure 4:**
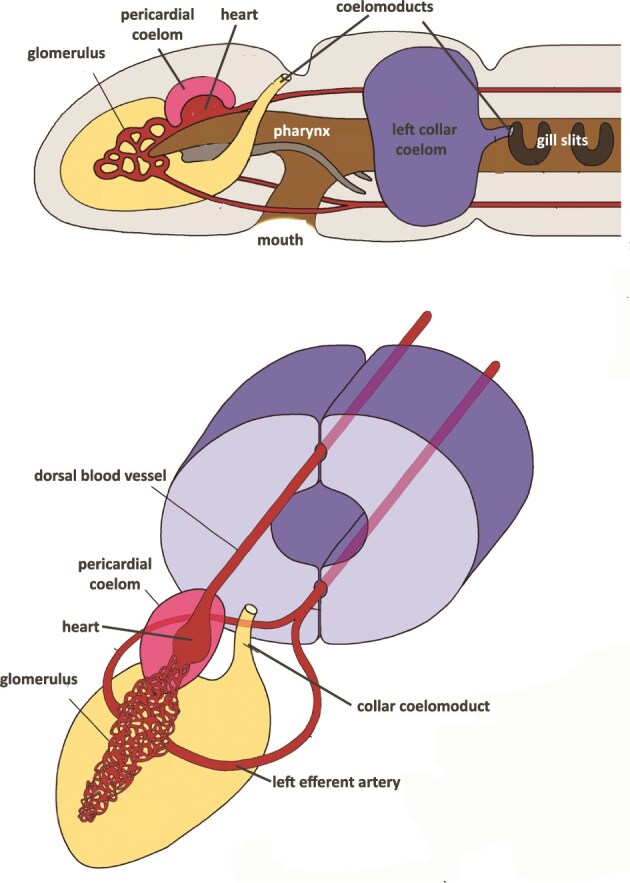
Views of the heart-kidney complex of later bilaterians (hemichordates), now known to be homologous with successive chordates and vertebrates. Protonephridial coelomoducts have now become gill slits and communicate directly from the exterior to the coelomic cavity (pharyngeal gut) for filter-feeding. More rostrally, protonephridial ducts lose their external openings and drain filtrate from the interstitium to an arterial anastomosis, the glomerulus, through which filtrate passes into a single long mesonephric duct to be voided (not shown). Above: Sagittal view of heart-kidney complex in the hemichordate acorn worm *Saccoglossus*. Below: Left anterior view of the asymmetrical arrangement of the left proboscis coelom enclosing the simple glomerulus, homologous with the chordate glomerular capsule (of Bowman). Cranio-caudal folding in the human embryo will bring the heart and segmenting mesonephros caudally into the trunk (see Fig. 5). Figure adapted from [39].

### The mesonephros

About 635 million years ago, later bilaterian (hemichordate, also termed ‘deuterostome’) ancestors of humans evolved the mesonephric (‘middle’) kidney. We define the mesonephros as that part of the primordial kidney retaining a connection to its longitudinal duct and located caudal to the pharynx. In contrast to the ‘fore-kidney,’ which became incorporated into the alimentary system, these more caudal portions of the primitive pronephros remained in the urinary system. They lost their external connections through the body wall but retained their internal connections to the longitudinal duct that was now renamed the mesonephric duct. This duct passed dorsal to the gut and entered the tail end of the coelom to exit the body at the cloaca, with the evolving cardiovascular system developed dorsal to the coelom. The membrane-lined body cavity, the coelom, surrounding the now enlarged gut, became the terminus of the mesonephric duct, exemplified by such extant hemichordate taxa as acorn worms [32] (Table 1, Fig. 4).

Ancestral hemichordates had a primitive hemolymph system draining the interstitial fluid space of the body that parallelled the developing arterio-venous circulatory system. Afferent lymphatic vessels maintained a vascular connection to the lower-pressure venous drainage, returning fluid to the circulation. The thoracic and left lymphatic ducts emptying into the left subclavian artery in humans exemplify this pattern. Efferent lymphatic channels, however, disappeared under the influence of higher-pressure mesonephric arteries coming from the heart. Mesonephric nephrons closed off their serial outlets to the exterior and drained urine instead into the remnant of the pronephric duct, now renamed the mesonephric duct. The mesonephros had a greater glomerular filtration rate, more filtering capacity, and more rapid flow through larger diameter ducts. Hemolymph from the interstitium became a component of plasma in the blood and was pushed under pressure to dispersed glomeruli, in which ultrafiltration occurred (Fig. 4).

The flow of urine in the pronephros was driven by ciliary action from the interstitial fluid space to the exterior. In the mesonephros, interstitial fluid was pulled into the venous and lymphatic systems by osmotic pressure and filtered through the glomeruli by hydrostatic pressure generated by the heart. Dorsal portions of the coelom fragmented into many isolated spaces as double mesothelial-lined pockets were pinched off to become the future glomerular (Bowman’s) capsules, into which the glomerular capillaries invaginated. The tail-end (‘opisthonephric’) mesonephros of the primitive vertebrate, the lamprey, preserves a single partially segmented ‘glomus’ that may represent an intermediate phase of glomerular capsule evolution [43] (Table 1).

Filtrate from the glomerulus flows into the collecting ducts and thence into the ureter of the future metanephros, which is a derivative of the ureteric bud, a caudal mesonephric duct in turn developed from the lowest pronephric duct. This duct develops to transmit urine from kidney to bladder because the middle and cranial portions of the mesonephric (Wolffian) ducts were preempted by the reproductive system to form the ductus deferens to transmit semen [44] (see Explanatory Box 2). The urethra is a tube that runs from the bladder to excrete urine to the exterior of the body. It is a derivative of the embryonic urogenital sinus [36].

Box 2. An Explanation of the Male ‘Wolffian’ Ducts and the Female ‘Mullerian’ Ducts

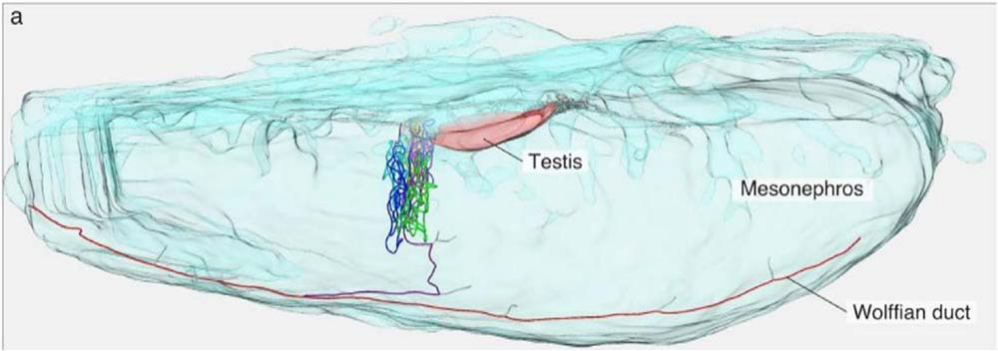

Teasing apart the Evo-Devo details of the evolution of the kidney has also elucidated important aspects of the origins of the male and female reproductive systems, which share a developmental site of origin in the nephrogenic ridge of the intermediate mesoderm. The mesonephric duct in the XY human embryo is coopted from the urinary system by the reproductive system to become the adult duct that delivers sperm to the ejaculatory duct and on to the exterior of the body. Long known as the ‘Wolffian duct’ it was discovered by the early German-Russian embryologist Kaspar Wolff and shown to be the primordium of the ductus (vas) deferens. Initially it was thought that the ‘Wolffian’ duct was also homologous to the female uterine (fallopian) tube, but the German embryologist Johannes Müller demonstrated that an entirely different duct originating from the infolding coelomic wall led from the primitive oviduct opening to the region of the ovary, forming the uterine tube. This embryonic structure is known as the paramesonephric (‘Müllerian’) duct. In the above 3D reconstruction using immunohistochemical markers of a 10-day old embryonic male frog (*Xenopus*) Omotehara and colleagues [45] showed the left rete testis connecting to the mesonephric (Wolffian) duct. Craniad is to the right. The mesonephric (Wolffian) duct and mesonephric tubules are represented in red and other colors, respectively. Cord-like structures in gray represent the Pax2-positive rete (testicular) cells. Frogs diverged from the human lineage some 350 million years ago. At this time the proximal mesonephric duct directly connected to the rete testis and was captured by the male reproductive system in the tetrapods. The ductus (vas) deferens was formed from the remnant duct as the mesonephros itself was degenerating and the metanephros was evolving. This embryological history explains why the ductus, a mesonephric structure, bypasses the ureter, a separate metanephric structure entering the bladder, to enter the urethra, a derivative of the alimentary system distal to the bladder. Adult females have a mesonephric duct rudiment (Gärtner’s duct) lateral to the vaginal wall which occasionally can be the site of inflammation. This embryological history also explains the paramesonephric opening of the uterine tube in the female, the only such piercing of the peritoneal cavity in the human body and an important part of the etiology of intracoelomic ectopic pregnancy. Males retain a blind paramesonephric rudiment, the paradidymus.

The clinical relevance of this evolutionary scenario is that peripheral pitting edema, a side-effect of decreased hydrostatic pressure, in chronic kidney failure or heart failure, or reduced plasma colloidal osmotic pressure, results from fluid first backing up into the dependent parts of the body’s interstitial space, e.g. the feet, ankles, and hands. Only secondarily does interstitial fluid breach the coelomic membrane and flow into derivatives of the body cavity, the peritoneal cavity (resulting in ascites) or the lungs (resulting in pulmonary edema).

**Figure 5:**
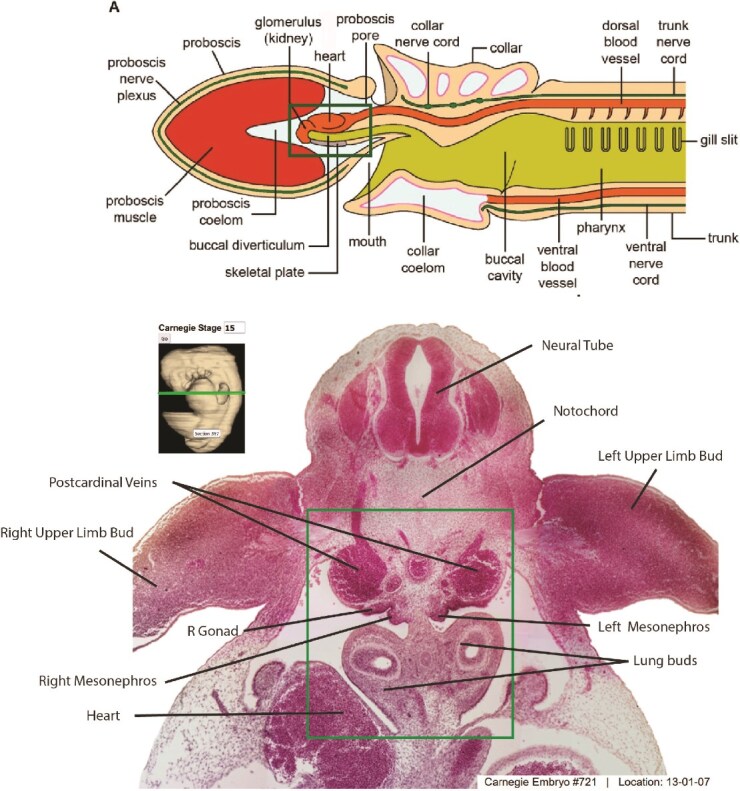
Top: Lateral view of a generalized ancestral hemichordate from [40] showing the close association the 1-chambered heart, the external glomerulus of a segment of the early mesonephric kidney, surrounded by the proboscis coelom, a possible homologue of the parietal wall of the glomerular capsule (of Bowman). The coelomoduct exits through the body wall. Below: A view from the caudal aspect of a human embryo at Carnegie Stage 15 (Week 7) showing the close spatial relationship of the mesonephros with the developing heart, a reflection of the embryological development from the pre-chordate heart-kidney. Image from [47]. Green boxes highlight the juxtaposition of heart and mesonephros in ancestral hemichordate and human embryo.

### The metanephros

Prior to the evolution of the metanephric kidney, the mesonephric kidney, coupled with a 2- chambered heart, was a successful excretory adaptation that lasted for over 150 million years, from early chordates to early fish, as exemplified by the extant lamprey (Fig. 1, Table 1). However, the mesonephric glomerulus was a lower-pressure filtration system that was superseded by the metanephros, with much higher glomerular filtration pressure, an adaptation that was foreshadowed in dipnotetrapodomorph lungfish in the Devonian Period (Fig. 1, Table 1). These remarkable vertebrates with mesonephric kidneys could adapt to the extremes of freshwater as well as aestivation in dry seasons. Urine flow rates and glomerular filtration rates (GFR) are high but ion excretion low compared to freshwater teleosts [46]. The environmental context in which the metanephros evolved included freshwater rivers, streams, and lakes: a large and diverse spectrum of ecological niches that had not been occupied by vertebrates.

The most revolutionary change in the long evolutionary history of the human kidney occurred when the limbless and still wormlike early chordates ventured from the ancient sea of their ancestors into new and uncharted territory—freshwater (Table 1). The medium was the same, but this water held an unknown and invisible danger. An over-zealous lancelet, dependent on its pronephros without the development of a tubular diluting segment, darting impulsively into freshwater after prey could suddenly find itself overcome and obtunded by water intoxication, a massive influx of fresh water into its mostly saline body tissues. Within minutes it would die if it could not swim back to its briny haven. ‘Water poisoning’ can still (rarely) affect humans who drink too much water during or after lengthy and strenuous exercise, such as running a marathon. Death can even result, and from basically the same reason that our ancient lancelet in the river succumbed. Exogenous freshwater pouring into the body can thereby dilute the blood and the interstitial fluid such that salt (sodium chloride) reaching the cells is not sufficient to drive the sodium-potassium pumps necessary for life. The lost lancelet and the water-intoxicated marathon runner both suffer from dilutional hyponatremia.

Exaptations existed, however, that were selected to raise the blood pressure, reinforce the glomeruli to withstand greater filtration pressures, and increase the metabolism to swim in swiftly running currents. The metanephric kidney, with its high pressure glomerulus and complex differentiated tubule to modify the filtrate was able to excrete the massive influxes of water that overwhelmed the mesonephros of their ancestors. Early vertebrates first populated the large rivers, but as species expanded their ranges into smaller streams and lakes, another major environmental obstacle confronted them. Unlike the sea and larger rivers, which were constant, smaller water bodies were subject to periodic drying out. The metanephric kidney responded by evolving mechanisms in which metabolism could continue even in air. Figure 5 shows the early differentiation of the lung buds, bilateral outpocketings of the tracheoesophageal diverticulum that form the air-breathing lungs, an adaptation that allowed aestivating lungfish ancestors to respire even as they lay dormant in desiccated lake beds awaiting the return of the rains. An increasingly complete Devonian fossil record has provided strong support for this hypothesis [40, 41]. Evidence for a freshwater transition from marine to terrestrial environment (as proposed by Homer Smith [6], and supported here) has been recently contested (See Explanatory Box 3).

Box 3. Controversy in the interpretation of the current phylogenetic-paleontological history of the mammalian kidney.

Homer Smith’s conclusion that the metanephric kidney owes its origins to an ancestor in a freshwater environment has been recently challenged by arguments for a marine rather than freshwater ancestry. Meyer and Hostetter [48] proposed that high GFR in the human kidney is an adaptation to excrete small molecular weight toxins present in a marine environment and correlates with increased metabolic rate rather than an ancestral need to excrete water in a hypotonic environment. Our analysis demonstrates that whereas marine invertebrates evolved ultrafiltering nephridia homologous with metanephric podocytes over 750 million years ago, evolution of an opisthonephric kidney did not develop until 200 million years later, reflected in extant primitive vertebrates with a two-chambered heart (lamprey) (Fig. 1 and Table 1). The metanephric nephron did not evolve until 250 million years later as amniotes adapted to a terrestrial environment with a 3-chambered heart, and another 180 million years before the appearance of homeothermic mammals with a 4-chambered heart, higher blood pressure, and increased metabolism coupled with increasing GFR. More recently, Evans [49] proposed a marine origin for the glomerulus in ‘first vertebrates,’ but it is based on misinterpretation of a report describing the divergence of fossil jawed fishes dated 480 to 360 million years ago, largely already radiating into marine habitats [50]. His claim contradicts the report of Devonian fossil lobefin fish from Greenland freshwater and floodplain sediments [51] and freshwater streams for Tiktaalik from Devonian sediments in Newfoundland [52]. Extant lobefin fish inhabit freshwater in Africa, South America, and Australia and fossil lungfish burrows reveal that lungfish freshwater habitats have persisted through the Mesozoic and Cenozoic eras [53, 54]. Moreover, early tetrapod fossils from the Devonian of the northeastern United States showed close association of early tetrapod fossils with flooded freshwater woodland environments [55]. There are many possible environmental factors that may have contributed selection pressure towards evolution of the metanephric kidney. However, accumulating evidence over the past 70 years has not diminished the importance or likelihood of Homer Smith’s contributions.

The glomerulus, a tuft of arterial capillaries that supplied blood to the small encapsulated coelomic space of the glomerular (Bowman’s) capsules, solved the ancient lancelet’s freshwater dilemma by vastly increasing its filtration rate [56]. The vertebrate kidney’s primary function, after waste removal, is to excrete water and excess ions while retaining the essential salts, nutrients, and blood proteins in the body [6]. The evolved vertebrate kidney differentiated the specific excretory (versus the more generalized emunctory) removal of nitrogenous wastes and water from the body. The glomerulus filters water and solute from the extracellular areas between organs and other structures in the body outside the cells themselves, and the tubule is responsible for fine-tuning of the final urine composition and volume by reabsorptive and secretory processes, critical to the selective excretion of water. The distribution of total body water has significant implications for the maintenance of homeostasis (See Explanatory Box 4).

Box 4. Evolutionary history clarifies the compartmentalization of human body fluids.

The anatomically disaggregated extracellular fluid volume (ECFV), composed of internalized ancient seawater (sodium-rich intercellular fluid and plasma), is now modified and maintained in diverse membrane-bound compartments which natural selection has adapted for specific functions in the vertebrate body. Blood, lymph, saliva, endolymph, perilymph, tears, sweat, milk, mucous, cerebrospinal fluid, and semen are all examples of sodium-rich derivatives of seawater which make up roughly one third of the body’s water. The intracellular fluid volume or ICFV comprises two-thirds of the body’s water inside the cells themselves, the high-potassium intracellular fluid [29]. The extracellular fluid space may be subdivided into the interstitial fluid volume (ISFV) and the plasma volume (PV). The colloquially used term ‘third space’ refers to that portion of the extracellular fluid that can accumulate in the interstitial space (as opposed to the intracellular and intravascular spaces) following surgical procedures, in chronic kidney disease, and in heart failure [57].

### Heart and kidney co-evolution

The heart and the kidney shared an intimate co-evolution (Figs 1 and 5) [39]. The single- chambered ‘heart-kidney’ was a pre-chordate adaptation that elevated the ultrafiltration pressure of the mesonephric glomeruli. The developing heart was connected with a diffuse set of capillaries supplying nearby glomeruli. The combined relationship of the pericardial coelom surrounding the developing heart, the adjoining anterior proboscis coelom, and the primordium of vascularized glomeruli, set the stage for the evolution of a single high-pressure excretory organ, the metanephric kidney (Table 1, Fig. 1). Selection pressures contributing to the evolution of the metanephric kidney have had a similar dramatic impact on interdependent organs. This includes the appearance of the four-chambered heart that increases oxygen delivery to tissues with complex capillary circulation, such as the kidney. Equally important, evolution of endothermy followed the innovation of nonshivering thermogenesis in brown adipose tissue and increased expression of uncoupling protein 1 (UCP1) [58]. This was a 2-step process beginning with the appearance of non-shivering thermogenesis in ectothermic teleosts over 400 mya, followed by the development of thermogenic UCP1 in placental mammals 180 mya. Endothermy is linked to increasing blood pressure that enhanced delivery of oxygen to highly metabolic organs including the brain, liver, heart, and kidney. Resulting increased glomerular capillary pressure in mammalian glomeruli led to development of stress fibers in extraglomerular mesangial cells with cytoplasmic processes adhering to the glomerular basement membrane. These adaptations maintain the integrity of the high pressure glomerulus [59], but fail in the face of hypertension or glomerular injury.

The evolutionary transition from hemichordate to chordate descendants is not clearly recorded in the fossil record. We must therefore rely on ontogenetic clues to reconstruct this history, which the molecular clock indicates took place approximately 588 million years ago (Table 1). Chordates evolved a closed circulatory system, i.e. one with arteries leading away from the heart, passing through a capillary ‘portal system’ through the glomeruli, postglomerular capillaries, then connecting through veins returning to the heart. The human embryo from Carnegie Stages 10 to 15 (weeks 3 to 7) shows a similar proximity of the developing heart to the intermediate mesoderm of the mesodermal ridge which forms the embryonic mesonephric kidney (Fig. 5).

This early spatial-functional relationship of the primitive heart and renal primordia was transfigured by subsequent development of the cardiovascular system and the metanephros, but the human lymphatic system is a remnant of this ancient pre-chordate adaptation. In humans the lymphatic system remains isolated from the high-pressure and ‘closed’ arteriovenous circulatory system, but lymph is eventually pushed into the venous system through the thoracic duct at the left subclavian vein by intra-abdominal and intra-thoracic pressure.

Box 5. Physiology of the urine concentrating mechanisms of the mammalian metanephros.

**Figure 6:**
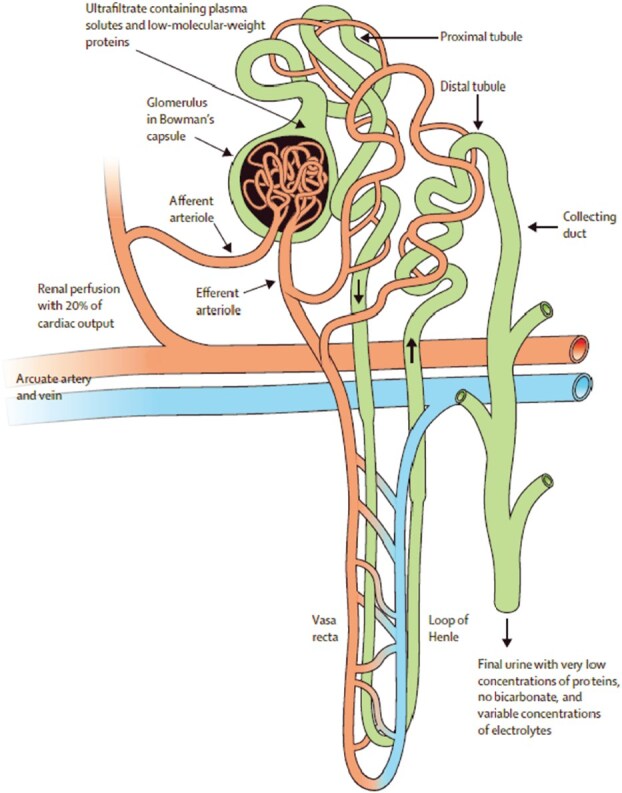
Structure of the mammalian metanephric nephron. A major anatomical change in the mammals was the loss of the renal portal system (the afferent arterioles do not enter an arteriovenous capillary bed from which veins emerge, but rather anastomose with glomerular arterioles). This adaptation reduces vascular resistance and allows a higher-pressure blood flow to the glomerulus, accounting for 20% of cardiac output. A second major adaptation of the mammalian kidney is the differentiation of functional segments of the aldosterone-sensitive distal nephron, comprising the distal convoluted tubule and collecting duct [64–66]. The distal convoluted tubule transports sodium, potassium, magnesium and calcium and functions as a diluting segment analogous to its origins in organisms adapting to a freshwater environment. Dilution of tubular fluid in the thick ascending limb and distal convoluted tubule is followed by passive movement of water out of the water-permeable cortical and medullary collecting duct in the presence of vasopressin. The connecting tubule and cortical collecting duct contain principal cells that regulate sodium reabsorption through ENaC channels in response to aldosterone, whereas intercalated cells (lacking primary cilia) mediate acid–base and potassium transport and sodium reabsorption through regulation of ENaC channels. The third major adaptation includes the loop of Henle, along with active sodium reabsorption from the thick ascending limb and urea recycling from collecting duct to vasa recta markedly enhance urine concentration through the countercurrent system. From [67] and by permission from *the Lancet*.

Due to differential transepithelial properties of the tubule segments, energy-dependent active transport of sodium chloride from filtrate to interstitium around the thick ascending limb promotes passive flow of sodium and urea (but not water) out of the thin ascending limb. This generates a corticomedullary osmotic gradient in the interstitium, concentration of filtrate in the collecting duct, and excretion of hyperosmolar urine. The length of the loop is a major (but not the only) determinant of maximal urine concentrating capacity, with a variable combination of long and short loops in mammals. Superficial cortical glomeruli are connected to short loops, and juxtamedullary glomeruli have long loops. Longer loops predominate in mammals adapted to arid environments (kangaroo rats) whereas shorter loops are more numerous in aquatic mammals (beavers). In contrast to birds, that excrete nitrogen as uric acid, mammals have boosted their capacity for water conservation by the incorporation of the arginine–urea cycle (AUC) in tubular metabolism. The AUC evolved in unicellular eukaryotes in response to variable available nitrogen in their marine environment (an anabolic process). The AUC was later adapted by metazoans in their catabolic metabolism of nitrogen for its excretion as urea. A hydrophilic molecule, urea can disrupt a protein’s hydrogen bonds that preserve its 3-dimensional folded configuration necessary for its function. This presents a challenge encountered by coelacanths excreting urea, and mammals relying on urea as a solute to amplify urinary concentrating capacity. Evolved production of methylamines enhance protein folding and ligand binding and counteract perturbation by urea [62]. In terrestrial mammals water conservation is accomplished by binding of arginine vasopressin (AVP) to V2 receptors on the inner medullary collecting duct that signal activation of urea transporters. By maintaining the osmotic gradient and increasing urea and water permeability this maximizes water conservation but also increases sodium reabsorption in the thick ascending limb, thereby reducing tubuloglomerular feedback and increasing glomerular filtration rate (Fig. 6). This is important, as high glomerular filtration must be matched by parallel energy-consuming reabsorption of sodium, glucose, and amino acids. In patients with severe burns, pathologic hyperfiltration results from high levels of AVP responding to fluid loss. This is augmented by greater recycling of urea generated by protein catabolism. The role of the kidney in adaptation to long-term fasting has followed divergent pathways through evolution [55]. Elephant seal pups undergo complete fasting for 2–3 months just after weaning. During aestivation in dry seasons, lungfish can survive 2–3 years encased in a subterranean cocoon with a small breathing hole [63]. Humans share a common ancestor with the seal 100 mya, and with the lungfish 400 mya. The lungfish markedly reduce metabolic rate and GFR, with activation of the vasotocin–aquaporin pathway, and generate water from protein catabolism. The seals maintain their high metabolic rate by not only activating vasopressin-aquaporin but also activating the aldosterone-ENaC pathway to conserve salt, and generating water by metabolism of stored lipids. By contrast, humans can only survive for 3–5 days without food or water.

### Adaptation to land

Transition to land presented a major challenge to an excretory organ that was adapted to a freshwater environment: rather than defending the organism from an influx of water, it became necessary to conserve water and sodium that could be scarce. The challenge became the matching of high GFR relative to the extracellular fluid volume. In addition, the interdigitated podocytes supporting the critical filtration barrier were vulnerable to increasing systemic blood pressure that threatened podocyte detachment and glomerular injury. This required finely balanced tubular adjustment of both extracellular fluid volume and sodium concentration in synchrony with autoregulation of renal blood flow that reduced barotrauma by maintaining intrarenal pressures at an optimal level [60]. The two primary mechanisms for renal autoregulation of GFR are myogenic (constriction of afferent and efferent arterioles mediated by the renin-angiotensin-aldosterone axis) and tubuloglomerular feedback (through signaling at the macula densa, juxataglomerular apparatus, and renin-angiotensin-aldosterone). Appearing in the early reptile lineage, these innovations maintained high GFR in the face of limited sodium intake. However, with current high human dietary sodium content, dysfunction of tubuloglomerular feedback or the juxtaglomerular apparatus now lead to hypertension [61].

Another key adaptation was the intermediate segment between proximal and distal segments in the mesonephric kidney of fish which became the loop of Henle in birds and mammals. This gave rise to the formation of a countercurrent multiplier system based on the juxtaposition of descending and ascending vasa recta and limbs of the loop of Henle (Fig. 6) (See Explanatory Box 5).

Bichirs, African eel-like freshwater fish that have functioning lungs can survive on land where they ambulate by using pectoral fins to propel themselves. Unlike most ray-finned fish that either remained in freshwater or transitioned back to a marine environment, bichirs evolved traits permitting survival in terrestrial environments. In an ingenious experiment comparing terrestrial- and aquatic-reared bichirs, the former walked faster and more efficiently, with morphology of neck and shoulder bones that parallelled those of lobe-finned fishes that evolved into amphibians [68]. This study revealed that environmentally induced phenotypes could be observed in ‘terrestrialized’ individuals, consistent with phenotypic plasticity likely of importance in the adaptations of stem tetrapods like lungfish. Ultrastructure of mesonephric nephrons of bichirs along with those of lungfish and amphibians is consistent with convergent adaptation to a terrestrial environment [69]. These studies support the relevance of an Evo-Devo approach to understanding the factors contributing to transition from a freshwater to a terrestrial environment.

## EVOLUTIONARY MEDICINE OF KIDNEY DISEASE

Evolutionary medicine focuses on understanding the ultimate etiologies of disease or pathology and formulating therapies that are populational, preventive, and usually long-term, whereas biomedicine primarily focuses on understanding proximate etiologies and formulating therapies that are individualized, reactive to internal or external environmental stressors, and usually short- term [22, 23]. Nevertheless, both approaches are fully compatible, and when used together, provide a more complete picture of a patient’s condition. Critical to our analysis was reliably ascertaining the geological (absolute) ages of adaptations of the kidney in human phylogeny.

This was undertaken with the realization that traits or structures when they first appear in evolution, albeit homologous based on comparative anatomy and genomic synapomorphies, are more generalized than their later, more derived and specialized forms. For example, the filtration apparatus of the renal glomerulus, although quite ancient (Proterozoic) in its single-cell origin as a flame cell, became a much more selective and specialized multicellular structure over time.

Another caveat is that there is an inverse relationship of the age of an adaptation and how fundamental it is in underlying the etiology of disease. For example, cilia represent an ancient adaptation that originated in single-celled choanoflagellates some 1.6 billion years ago.

Ciliopathies can manifest as such urinary-system-specific maladies as cystic kidney disease affecting the human kidney, yet this organ did not evolve to its current form until some 180 million years ago. Ancient adaptations can manifest as pathologies involving ciliary action affecting a wide array of systems in addition to the urinary system [70].

The evolutionary history of the mammalian nephron traced in this review can account for many of the vulnerabilities of the kidney to stressors that can occur in early life resulting in impaired nephrogenesis, or environmental stressors through aging and senescence [4, 5]. Urine production by the fetal metanephros begins at week 10, increasing exponentially with nephrogenesis through second and third trimesters, contributing to 50% of amniotic fluid volume. Because lack of metanephric development (bilateral renal agenesis) leads to impaired fetal lung development, infants born with this condition often die of respiratory failure shortly after birth. Throughout postnatal life, the high pressure-flow glomerulus predisposes podocytes to injury and detachment leading to sclerosis, whereas the high mitochondrial activity of the tubule contributes to susceptibility to ischemia, hypoxia, and oxidative injury. With ongoing nephron loss through the life cycle, overload of remaining nephrons has been proposed as a final common pathway for progressive kidney disease [71]. Hemodynamic overload is responsible for shear stress on the glomerular filtration barrier, whereas metabolic overload drives tubular injury [72]. The process of progressive nephron loss through the life span occurs through aging and contributes to hemodynamic and tubular damage that lead to acute kidney frailty [68]. These considerations harken back to the interdependence of the cardiopulmonary and renal systems that evolved hundreds of millions of years ago.

We have focused on the evolving primary excretory functions of the kidney in an empirically falsifiable ontophylogenetic framework intimately tying anatomy and physiology to time and environment. As Dawkins [73] has recently observed in supporting such an adaptationist paradigm,” the whole body through and through, its very warp and woof, every organ, every cell and biochemical process, every smidgen of any animal, including its genome, can be read as describing ancestral worlds.” An evolutionary medical corollary of this approach is that complex etiologies of renal pathologies involving many time-constrained genomic, cellular, tissue, organ, and inter-organ adaptations, within the contexts of past environments, are to be expected, and indeed required, in diagnosing ultimate causes.

Biomedical physiologists [29] have operationally delineated six discrete functions of the human kidney in addition to the primary function of excreting metabolic wastes and poisonous substances: (i) glucose synthesis; (ii) regulation of water and electrolyte balances; (iii) regulation of arterial pressure; (iv) regulation of acid–base balance; (v) regulation of erythrocyte production; and (vi) regulation of 1,25 dihydroxyvitamin-D3. It remains for ongoing and future research, such as genomic ‘multi-million-year natural experiments’ [74] aimed at understanding the sequence, timing, and causal interactions of these evolutionary adaptations, to unravel more fully the natural history of kidney disease. This will require approaches complementary to the Zoonomia genomics project, to include evolutionary physiology, developmental plasticity, and epigenetics [75–77]. Evolutionarily informed and more precise clinical diagnoses will ultimately enhance the quality of medical care.

## Supplementary Material

Glossary_eoaf019(1)
