# Supplementary material for: Evolutionary medicine of emunctory functions of the kidney: an empirical review
Source: Evol Med Public Health. 2025 Aug 5;13(1):229–47. doi: 10.1093/emph/eoaf019 (PMC12409785; doi:10.1093/emph/eoaf019)
Supplement: Glossary_eoaf019(1) [file glossary_eoaf019(1).docx]

Glossary

| AVP | Arginine vasopressin, also known as vasopressin and antidiuretic hormone (ADH), is a hormone secreted by the posterior lobe of the pituitary gland that constricts blood vessels, raises blood pressure, and reduces excretion of urine |
| --- | --- |
| Bilateria | Animals that develop with a bilateral body plan, usually with a differentiated head region with sensory adaptations |
| Chordate | Animals which possess a notochord (a stiff supporting rod in the dorsal midline of the body), a tail, a hollow nerve cord, gill slits opening from the pharynx to the exterior, and an endostyle (homolog of the thyroid gland) |
| Coelom | The definitive body cavity derived from and surrounded by mesoderm formed by growth and midline fusion of the body walls during craniocaudal folding of the embryo; a defining characteristic of coelomate animals |
| Coelomoduct | Duct extending along the dorsal body wall from the gonad through the coelom and out to the exterior of the body; the primordium of the paramesonephric (Mullerian) ducts, developing into the uterine tubes, uterus, and superior vagina in females and largely degenerating in males, forming only the rudimentary “appendix testis;” |
| Collecting Tubule of Metanephros (TE 3741) | The most distal part of the nephron between the nephron loop and the collecting duct |
| Deuterostome | Bilaterian animals whose embryonic blastopore becomes the anus, possessing a coelom and organs suspended within the body by mesenteries |
| Distal metanephric tubule (TE 3753) | Convoluted tubule of the metanephric nephron between the nephron loop and the collecting tubules |
| ECM | The extracellular matrix, a meshwork of proteins and other molecules that surrounds and supports cells within tissues and organs, with a role in regulating cell behavior, including growth, migration, and differentiation |
| Glomerular Capsule (TE 3759) | The cellular envelope into which filtrate from the glomerulus empties, formed internally by the endothelial walls covered with podocytes and externally by the coelomic wall, into which the glomerulus invaginates during development; also termed “Bowman’s Capsule” |
| Glomerulus [Metanephric] (TE 3761) | A tuft of capillaries between the afferent and efferent arterioles of the renal artery which invaginates a segment of the coelom to form a high-pressure filtering mechanism of blood which retains large molecules in the plasma and excretes water and small molecules into the urine and exhibits charge selectivity each glomerulus is located in the renal cortex within multiple metanephric renal lobes; glomeruli near the medulla empty into nephrons with a longer nephron loop |
| Homology | Similarity in structure or function in an organism that is due to shared ancestral descent and genetic determination |
| Intermediate Mesoderm (TE 3690) | A narrow section of the mesoderm (one of the three primary germ layers) located between the paraxial mesoderm and the lateral plate of the developing embryo, sometimes preferentially termed intermediate mesenchyme |
| Interstitium | The space within the body and inside its organs that exists between the cells, filled with extracellular (interstitial) fluid; the urinary system drains and filters this fluid which contains water and waste products from the body |
| Mesonephric Duct (TE 3699) | An epithelial tube, also termed the nephric, or Wolffian, duct, formed by mesenchymal-to-epithelial transformation from the intermediate mesoderm into a linear group of cells; primordium of the ductus (vas) deferens and the ureteric bud (from its most distal portion); mutually induces with the metanephrogenic blastema the metanephric kidney |
| Mesonephric Glomerulus (TE 3711) | A tuft of capillaries between the afferent and efferent arterioles of the renal artery which invaginates a segment of the coelom to form a low-pressure filtering mechanism of blood which retains large molecules in the plasma and excretes water and small molecules into the urine; each glomerulus is located in a single mesonephric renal lobe and empties into a nephron lacking a nephron loop |
| Mesonephros (TE 3696) | The “amphibian kidney,” a modular excretory organ with a glomerulus developing caudal to the pronephros, running bilaterally parallel to the vertebral column in tetrapods |
| Metanephridium | An advanced nephridium complex that extends from the interstitial fluid space to the exterior of the body with a connecting tubule from the coelom, characteristic of deuterostome animals or their embryonic development; When a coelomic cavity is present the inner end of the metanephridium tends to project through the coelomic epithelium into the cavity, and to open into it by a ciliated nephridiostome |
| Metanephrogenic Blastema (TE 3742) | Identifiable mass of rapidly developing, proliferating, undifferentiated cells of the intermediate mesoderm that gives rise to the glomerulus, tubules, and collecting ducts of the metanephric kidney; it is induced to develop embryonically by the ureteric bud at the most distal portion of the mesonephric duct; also termed the metanephric blastema |
| Metanephros (TE 3712) | The “mammalian kidney” or definitive human kidney developing from the ureteric bud and metanephrogenic blastema (primordia of glomerulus, proximal and distal convoluted tubules, and nephron loop) |
| Nephridium | Fine tubular structure that extends from the interstitial fluid space to the exterior of the body in animals, with a connection to the coelom in deuterostomes |
| Nephron (TE 3749) | Anatomical and functional unit of the kidney, consisting of the renal corpuscle, convoluted tubules, and collecting tubule |
| Nephron Loop (TE 3754) | Loop of Henle, a water-conserving part of the nephron characteristic of mammals; it constitutes a countercurrent multiplier running through the hypertonic interstitial space of the inner medulla, a configuration that is essential for the excretion of concentrated urine; a heterogenous segment, comprising the pars recta of the proximal tubule, the thin descending and ascending limbs, and the medullary and cortical thick ascending limbs; the thin descending limb is highly permeable to water, but not to salt, due to concentrated Aquaporin 1 in its epithelium. Trapping of potassium by countercurrent exchange between the ascending and descending limbs occurs in the medulla; the thick ascending limb extends towards the glomerulus of the same nephron and then leads into the distal convoluted tubule |
| Nephrostome | A funnel-shaped and ciliated orifice of an excretory tubule draining the interstitial space and opening into the coelom; characteristic of simple deuterostome animals |
| Nephrotome (TE 36920) | A segmented section of intermediate mesoderm developing between the somites medially and the lateral plate mesoderm laterally that gives rise to the urinary system |
| Podocyte (TE 3763) | Cells with foot processes (pedicels) that wrap around capillaries that connect the afferent and efferent arterioles in the glomerulus; podocytes form the visceral layer of the glomerular (Bowman’s) capsule and form the filtration slits that allow water and small molecules to flow out of the blood while retaining red blood cells and larger molecules in the plasma |
| Primordium (TE 29) | The first discernable indication of an organ or structure in embryological development; an embryonic structure that makes up part or all of a differentiating structure of adult form; the German “anlage” is a synonym |
| Pronephros (TE 36981) | The primordial kidney formed in the Neoproterozoic Era from intermediate mesoderm and primitively extending the length of the body; embryologically, formed during human Carnegie Stage 10 (Day 20) as the ephemeral “fore kidney” whose protonephridial slits are primordia of the gill slits of chordates |
| Protonephridium | A primitive nephridium formed by distinct filtering, absorptive, and channeling cellular segments draining interstitial fluid and cellular waste products from the parenchyma of the body out through openings in the body wall; a ciliated excretory canal opening to the exterior by a nephridiopore ending internally and blindly in flame cells or solenocytes |
| Protostome | Bilaterian animals whose embryonic blastopore becomes the mouth; refers to small bilaterally symmetrical worm-like animals without an internal body cavity, known as a “coelom.” Protostomes have a dedicated excretory system and are included by some in the paraphylrtic taxon Nephrozoa, along with animals with a coelom, e.g. deuterostomes. |
| Renal Corpuscle (3758) | The glomerulus and glomerular capsule; also known as the Malpighian corpuscle |
| Stroma of Kidney (TE 3743) | The parenchema or ground substance of the kidney containing the interstitial fluid space |
| TCA | Tricarboxylic Acid Cycle, also known as the Krebs Cycle or the Citric Acid Cycle; a series of chemical reactions using oxygen to produce adenosine triphosphate (ATP), the energy currency of the cell. |
| Ureteric Bud (TE 3715) | Primordium of the renal calyces, renal pelvis, ureter, and trigone of the bladder; the metanephric diverticulum |
| Vertebrate | Animal with a backbone and a mesonephric kidney at some phase of development; first animals with bones, parathyroid glands, respiratory gills, limb-like appendages, post-anal tail, and cranial nerves |
| Opisthokont | A broad group of eukaryotic organisms that includes animals and fungi, characterized by a flagellum on the rear (Greek “*opísthios”)* pole (Greek *kontós)* of the organism, similar to a human sperm cell; generally recognized as a clade, diverging some 1.3 billion years ago; choanoflagellates are an extant organismal homologue |
| Pseudocoelom | Space inside the body that is a remnant of the embryonic blastocyst cavity in acoelomate organisms; a space internal to the body wall not enclosed by an epithelium of mesodermal origin (a “mesothelium”) and lacking mesenteries |
| Rudiment (TE 471) | Underdeveloped or immature part or organ (Latin “that which is unwrought”); a remnant of an embryonic structure (a “rest”) that is retained in an adult; used preferentially ontogenetically, e.g. the appendix of the testis is a male rudiment of the paramesonephric duct |
| Ultrafiltration | Filtration through a medium, such as a semipermeable membrane, which allows small molecules, as of water, to pass, but holds back larger ones, as of protein; Materials which pass through the filter are referred to as “filtrate.” Materials which are held back or retained are referred to as the “retentate.” |
| Vestige (TE 32) | Body part or organ that has become reduced in function and/or in size in the course of phylogeny (Latin for “footprint”); structures that are remnants of organs or features that were fully developed and functional in earlier species, but may be no longer of benefit to the organism, e.g. the auriculares muscles of the human ear |

TE = Numbered term from Terminologica Embryologica (<https://libraries.dal.ca/Fipat/te2.html>).
